# An Episomal Clustered Regularly Interspaced Short Palindromic Repeats/Cas9 System for Transgene-Free Multiplex Gene Editing in Pig Cells

**DOI:** 10.3390/biology15100742

**Published:** 2026-05-08

**Authors:** Chaoqian Jiang, Dongyan Yang, Chengbo Sun, Xingrui Ren, Tianze Li, Jiayan Wu, Jian Tian, Mingjie Feng, Yuchang Yao, Jun Song, Xiaogang Weng, Yanshuang Mu

**Affiliations:** 1 Key Laboratory of Animal Cellular and Genetic Engineering of Heilongjiang Province, Northeast Agricultural University, Harbin 150030, Chinasongjun@neau.edu.cn (J.S.); 2 College of Life Science, Jilin University, Changchun 130012, China; 3 College of Animal Science and Technology, Northeast Agricultural University, Harbin 150030, China

**Keywords:** pig, CRISPR/Cas9, multiplex gene editing, SMAR

## Abstract

Despite significant advancements in CRISPR/Cas-based genome editing technology over the past decade, achieving simultaneous homozygous gene editing at multiple targets in primary cells remains a major challenge. In this study, we developed and constructed a CRISPR multi-gene targeting system that integrates episomal vectors with tRNA–sgRNA array technology. This approach leverages scaffold/matrix attachment region (S/MAR) sequences to enable sustained episomal expression of both Cas9 and single-guide RNAs (sgRNAs) without genomic integration, thereby enhancing gene editing efficiency. For simultaneous editing of multiple loci, we used the tRNA–sgRNA architecture to process multiple sgRNAs from a single vector. Using this system in porcine fetal fibroblasts, we achieved concurrent editing of six genes, namely *ANXA7*, *GSK3A*, *ENTPD6*, *SIRT3*, *CYP20A1*, and *SOCS2*, in individual cells. These edited cells supported normal development following somatic cell nuclear transfer, yielding blastocysts with unaltered developmental competence. Collectively, our findings establish a framework for the application of CRISPR/Cas9 in gene-edited pigs, facilitating the generation of multi-gene-edited animals for biomedical and agricultural applications.

## Simple Summary

Simultaneous CRISPR/Cas genome editing of multiple genes in primary cells remains a major challenge despite advances in genome editing technologies. Here, we developed a multi-gene targeting system that combines a non-integrating DNA vector with a strategy enabling the generation of multiple guide molecules from a single construct. This design supports the sustained expression of gene editing components without insertion into the host genome, thereby improving efficiency. In porcine fetal fibroblasts, the system enabled concurrent editing of six genes within individual cells. These edited cells retained normal developmental competence when used for embryo reconstruction. Overall, this study provides an efficient and reliable approach for generating animals with multiple precise genetic modifications, with potential applications in biomedical research and agriculture.

## 1. Introduction

Gene-edited pigs hold tremendous potential for both agricultural and biomedical applications [1]. In agriculture, gene-edited pigs have been utilized to enhance the production of high-quality meat and bolster disease resistance [2,3]. In biomedicine, pigs carrying targeted mutations in disease-related genes serve as invaluable large animal models for elucidating disease mechanisms and exploring therapeutic strategies for human genetic disorders [4].

The rapid evolution of the clustered regularly interspaced short palindromic repeats (CRISPR) system has left an indelible mark on the generation of gene-edited pigs [5]. Derived from the adaptive immune system of bacteria, CRISPR functions as a programmable endonuclease that enables precise manipulation of cellular genomes [6]. The most widely used variant, *Streptococcus pyogenes* Cas9 (SpCas9), is directed by a dual guide RNA comprising tracrRNA and crRNA to generate site-specific double-strand breaks (DSBs). These breaks are subsequently repaired through non-homologous end joining (NHEJ) or homology-directed repair (HDR), facilitating targeted gene modifications. A prevalent strategy for generating gene-edited pigs involves the use of primary cells harboring defined genetic alterations [6].

Specifically, porcine fetal fibroblasts (PFFs) are edited via Cas9/single-guide RNA (Cas9/sgRNA) ribonucleoprotein complexes or plasmid delivery, and clonal populations carrying the desired modifications are then used as nuclear donors for somatic cell nuclear transfer (SCNT) to produce live animals [1]. However, SCNT faces significant challenges when multiple genetic modifications are required. To produce pigs with multiple genetic modifications, multiple rounds of cloning are typically required, often using cells from previously cloned gene-edited pigs for additional gene editing. This iterative process is not only arduous and time-intensive but also risks the accumulation of epigenetic abnormalities, thereby compromising the overall efficiency in breeding pigs with multiple genetic modifications.

Theoretically, multiplex genome editing is achievable by using Cas9 in conjunction with multiple guide RNA (gRNA) targeting different target sites [7,8,9]. Microinjection or transfection of gene constructs containing multiple sgRNA-expressing cassettes into cells is two conventional strategies for multiple genome editing [8,9]. However, direct microinjection of Cas9 mRNA (or Cas9 protein) together with in vitro-transcribed gRNA into embryos or cells is feasible only in a limited number of species [10]. Another strategy entails the concatenation of multiple sgRNA-expressing cassettes [11]. Each cassette generally spans 400–500 base pairs and comprises an RNA polymerase III (Pol III) promoter, the sgRNA sequence, and a Pol III terminator. Due to limitations in delivery methods and the cargo capacity of plasmid vectors, the concurrent delivery of numerous sgRNAs into PFFs via this method remains challenging. To generate multiple gRNAs from a single transcript, tRNA processing technology has been developed. Multiple gRNAs have been generated from a single transcript through an extremely conserved tRNA processing mechanism. From an engineered gRNA–tRNA array (GTR), multiple gRNAs are expressed in tandem. Endogenous RNase P (a ribonucleoprotein) and RNase Z (a protein enzyme) recognize the tRNA moieties and cleave the array precisely, thereby releasing tRNA and multiple gRNAs [12]. This tRNA-based multiplexing approach has enabled efficient multi-gene editing across diverse plant, animal, and microbial species [11,13,14].

Despite the precision afforded by CRISPR-based targeting, a persistent limitation of current delivery systems is the risk of unwanted genomic integration of vector components [15]. Such integrated vectors can lead to insertion mutations, unpredictable genomic disruption, and irreversible changes. Nonviral, non-integrating vectors capable of sustained episomal persistence and long-term expression offer a promising alternative [16]. Among these, scaffold/matrix attachment region (S/MAR)-based episomal vectors have been widely investigated. S/MARs are cis-acting DNA elements that mediate the attachment of chromatin to the nuclear scaffold or matrix [17] and are ubiquitously present in all eukaryotes [18]. Although S/MAR sequences lack strict primary sequence conservation, their conserved secondary structural features, including replication origins, A+T-rich sequence motifs, MAR characteristics, and Topoisomerase II binding sites, are critical for their function. These elements typically span 300 to 3000 base pairs (bp) [18]. Among the S/MAR sequences described to date, the sequence corresponding to the 5′ region of the human interferon-β gene (hIFN-S/MAR) has been extensively studied and used to generate plasmids with replication capability [19]. Plasmids harboring hIFN-S/MAR exhibit mitotic stability, persisting episomally through multiple cell divisions in the absence of sustained selection [20]. Compared with integrating viral vectors, S/MAR-based vectors offer distinct advantages, including larger cargo capacity and lower treatment-related toxicity. Pioneering studies have demonstrated their utility in stably engineering therapeutic genes into hematopoietic progenitor cells [21,22] and achieving durable gene correction in murine models of blindness [23].

In this study, we developed a high-capacity, multi-gene episomal CRISPR/Cas platform for multiplex modification of the porcine genome (the GTR-CRISPR system). This system harnesses the synergy between tRNA–sgRNA array technology and S/MAR-based episomal vectors, enabling sustained extrachromosomal maintenance and prolonged Cas9/sgRNA expression without genomic integration, thereby enhancing editing efficiency at multiple loci. To verify the editing efficiency of the gene editing system, this study selected six genes as targeted genes, including *ANXA7*, *GSK3A*, *ENTPD6*, *SIRT3*, *CYP20A1*, and *SOCS2*. These genes are related to muscle development, fat deposition, and growth rate [24]. Our findings demonstrate that the CRISPR–S/MAR system can be designed as a flexible tool for effective multiplex genome editing in pigs and facilitates the comprehensive analysis of multigenic regulatory networks. This strategy thus offers a powerful approach for livestock genome engineering, with broad applications in agricultural and metabolic biotechnology.

## 2. Materials and Methods

### 2.1. Plasmid Vector Construction

pCas9GS: The episomal targeting vector mainly consists of four functional modules: the sgRNA domain, the Cas9 domain, the SMAR domain, and the G418 domain. SMAR is derived from P-EGFP-SMAR (Cat. No. V006530, Newpu Biotechnology, Shanghai, China), and G418 is derived from Cas-G418 (addgene Cat. No. 60847, Watertown, MA, USA). It is constructed with PX330-U6-sgRNA-Cas9 (addgene Cat. No. 223323, Watertown, MA, USA) as the backbone. A 9134 bp fragment from the pEGFP-SMAR (Cat. No. V006530, Newpu Biotechnology, Shanghai, China) vector is amplified by PCR as the vector backbone, a 168 1bp G418 fragment (addgene Cat. No. 60847, Watertown, MA, USA) with homologous arms is amplified, and a 1226 bp AMP fragment (addgene Cat. No. 98290, Watertown, MA, USA) with homologous arms is amplified. The three fragments are ligated by seamless cloning with HiFi DNA Assembly Master Mix (NEB Cat. No. E2621S, Ipswich, MA, USA) to generate the final construct pCas9GS (PX330-U6-sgRNA-Cas9-GFP-SMAR).

In this experiment, the constructed episomal vector pCas9GS was used as the backbone, which carries the U6 promoter and the sgRNA scaffold sequence. To facilitate the subsequent insertion of sgRNA, the vector was digested at the BbsI (NEB Cat. No. R3539S, Ipswich, MA, USA) restriction site.

To carry out functional verification experiments, a control plasmid without the SMAR fragment was constructed: the SMAR fragment was excised from the pCas9GS-ANXA7 plasmid, and then the vector backbone was religated to obtain the control plasmid pCas9G-ANXA7.

pCas9GS-6sg: With the pCas9GS vector as the backbone, the above sgRNAs were linked into a single sequence via tRNA sequences and synthesized commercially as 6sg-tRNA. This fragment was inserted into the pCas9GS backbone by HiFi DNA Assembly Master Mix (NEB Cat. No. E2621S, Ipswich, MA, USA) cloning to generate the pCas9GS-6sg vector.

Cas9-GFP-G418: A Cas9 plasmid with Neo resistance.

### 2.2. Design sgRNA

Locate the exon region of the target gene on NCBI, then design sgRNA sequences via the CRISPOR website (https://crispor.gi.ucsc.edu/crispor.py (accessed on 5 May 2026)) and select the sequences with high scores. The sgRNA sequences are shown in Supplementary Table S1.

In the sgRNA design step, six target genes related to pig breeding (*SOCS2*, *ENTPD6*, *GSK3A*, *SIRT3*, *ANXA7*, and *CYP20A1*) were selected. sgRNAs were designed, targeting the exon regions of each gene. After screening for sgRNAs with high scores, complementary upstream and downstream sgRNA sequences of 20 bp in length with sticky ends were synthesized (see Supplementary Table S1 for detailed sequences); meanwhile, PCR primers used for amplifying each target gene were designed (see Supplementary Table S2 for detailed primer sequences). The synthesized sgRNA oligonucleotides contained a 5′ CCGG overhang at the upstream end and a 5′ AAAC overhang at the downstream end. The synthesized sgRNA sequences were inserted into the pCas9GS vector digested with BbsI, and finally, six targeting episomal plasmids were constructed, which were named pCas9GS-SOCS2, pCas9GS-ENTPD6, pCas9GS-GSK3A, pCas9GS-SIRT3, pCas9GS-ANXA7, and pCas9GS-CYP20A1.

### 2.3. Cell Culture

Primary porcine fetal fibroblasts (PFFs) were isolated from 33-day-old Large White pig embryos. Embryos were isolated and washed and dorsal muscle tissue was collected, minced, and digested with 0.1% collagenase followed by 0.25% trypsin. Cells were collected by centrifugation, seeded into culture dishes, and maintained at 37 °C with 5% CO_2_, with the medium changed every other day. Passage 1 cells were cryopreserved using a stepwise cooling method. All animal procedures were approved by the Institutional Animal Care and Use Committee (IACUC) of Northeast Agricultural University. Cryopreserved PEF cells were rapidly thawed in a 37 °C water bath with gentle agitation, resuspended in 500 μL of a medium containing 15% FBS, and centrifuged at 1200 rpm for 3 min. The supernatant was discarded, and the cell pellet was resuspended in fresh 15% FBS medium and subsequently seeded into culture dishes for further cultivation. PFFs were cultured in DMEM (Gibco, Cat. No. 12491015, Grand Island, NY, USA) supplemented with 15% FBS (FBS; Gibco, Cat. No. A5256701, Grand Island, NY, USA), 1% penicillin–streptomycin (CELLGRO, Cat. No. 30-002-CI, Medford, NY, USA), 1% nonessential amino acids (Invitrogen, Cat. No. 11140-050, Carlsbad, CA, USA), and 2 mM L-glutamine (Sigma, Cat. No. G8540-100G, St. Louis, MO, USA) at 37 °C with 5% CO_2_.

### 2.4. Cell Transfection and Selection

Approximately 1 × 10^6^ PEF cells were trypsinized, pelleted, and resuspended in 20 μL of Opti-MEM (Gibco, Cat. No. 31985-070, Grand Island, NY, USA) with 5 μg of the plasmid DNA. Electroporation was performed using the CUY21 electroporator (BEX, Cat. No. BEX CUY21 EDIT II, Ltd., Tokyo, Japan). The BEX electroporator was set as follows: poring voltage of 150 V, 20 ms, for 5 cycles; stabilizing voltage of 10 V, 10 ms, for 5 cycles. After 48 h, cells were subjected to G418 (0.8 mg/mL) (Sigma-Aldrich, Cat. G5013, St. Louis, MO, USA) selection for 3 days before harvesting.

### 2.5. Plasmid Rescue Assay for Verifying S/MAR Episomal Maintenance

Viral DNA was isolated from the screened cells after 14 days of culture using the EasyPure^®^ Viral DNA/RNA Kit (Cat. No. ER201-01, TransGen Biotech, Beijing, China). The purified DNA was subsequently transformed into chemically competent *E. coli* cells (DH5a Cat. No. DL1001, WEIDI, Shanghai, China), and the transformed bacterial suspension was spread evenly onto selective agar plates, followed by overnight incubation at 37 °C. After incubation, colony growth status was visually examined, and individual monoclonal colonies were randomly picked for Sanger sequencing to confirm the identity of the target DNA fragment.

### 2.6. Oocytes’ In Vitro Maturation

Pig ovaries from prepubertal gilts were collected at a local slaughterhouse and transported to the laboratory in an insulated container maintained at 37 °C. Antral follicles between 5 and 8 mm in diameter were aspirated manually with a disposable 10 cc syringe and an 18-gauge needle. Follicular fluid was pooled and allowed to settle by gravity. Cumulus–oocyte complexes (COCs) were resuspended in Hepes-buffered medium containing 0.01% polyvinyl alcohol (PVA). Under a dissecting microscope, COCs with multiple layers of intact cumulus cells were selected for the experiments. Around 50–75 COCs were placed in 500 µL of tissue culture medium 199 (Cat. No. TCM-199; Gibco BRL, Grand Island, NY, USA) containing 0.14% PVA, 10 ng/mL epidermal growth factor, 0.57 mM cysteine, 0.5 IU/mL pig FSH, and 0.5 IU/mL ovine LH. COCs were matured for 42–44 h at 39 °C and 5% CO_2_ in air and 100% humidity. All chemicals were obtained from Sigma Chemical Company (St. Louis, MO, USA) unless stated otherwise.

### 2.7. Production Cloned Embryos

Porcine oocytes matured in vitro were used as recipient oocytes for nuclear transfer. Following 42–44 h of maturation culture, the oocytes were treated with 1 mg/mL hyaluronidase (Sigma-Aldrich, Cat. No. 37326-33-3, St. Louis, MO, USA) to remove the surrounding granulosa and cumulus cells. Oocytes that clearly extruded the first polar body were selected as recipient cytoplasts.

Cumulus-free (denuded) oocytes were enucleated by aspirating the first polar body and adjacent cytoplasm using a glass pipette with a diameter of 25 μm. The enucleation was performed in TCM-199-Hepes medium supplemented with 0.3% bovine serum albumin (BSA, Sigma-Aldrich, Cat. No. V900933, St. Louis, MO, USA) and 7.5 μg/mL cytochalasin B (Sigma-Aldrich, Cat. No. 14930-96-2, St. Louis, MO, USA).

The donor cells used were double-screened monoclonal porcine embryonic fibroblasts (PEFs) that had undergone prior identification and validation. These monoclonal PEFs were injected into the perivitelline space of the enucleated oocytes. The injected oocytes were then transferred to the fusion/activation medium, which consisted of 0.3 M mannitol, 1.0 mM CaCl_2_, 0.1 mM MgCl_2_, and 0.5 mM HEPES.

Fusion and activation were induced using two direct current (DC) pulses of 1.2 kV/cm for 30 ms, delivered via a BTX Electro-Cell Manipulator 2001 (BTX, San Diego, CA, USA). After fusion/activation, the reconstructed embryos were cultured in porcine zygote medium 3 (PZM-3) at 38.5 °C in an atmosphere of 5% CO_2_ in air.

The cleavage rate was evaluated at 48 h post-activation, and the blastocyst rate was assessed at 144 h post-activation.

### 2.8. Single Blastocyst Lysis

On Day 7 post-fertilization, morphologically normal blastocysts were collected and washed three times in sterile phosphate-buffered saline (PBS) (Sigma, Cat. No. D8537-100ML, St. Louis, MO, USA) containing 0.1% polyvinyl alcohol (PVA) to remove any attached cells or debris. Individual blastocysts were transferred into sterile 0.2 mL PCR tubes containing 3–5 µL of the lysis buffer (0.5% Triton X-100 (Sigma-Aldrich, Cat. No. X100-100ML, St. Louis, MO, USA), 0.2 mg/mL Proteinase K (Takara, Cat. No. 9034, Tokyo, Japan)). Samples were incubated at 55 °C for 1 h to achieve complete lysis, followed by enzyme inactivation at 95 °C for 10 min. The lysates were then used directly as templates for PCR without any further DNA purification or amplification.

### 2.9. Genomic DNA Extraction and PCR

PCR conditions: 95 °C for 5 min and 35 cycles of denaturation at 95 °C for 30 s, annealing at 55 °C for 30 s, and extension at 72 °C for 30 s, followed by a final extension at 72 °C for 10 min. Genomic DNA was extracted using the Universal Genomic DNA Extraction Kit (Takara, Cat. No. 9765, Tokyo, Japan). Targeted fragments (200–300 bp) were PCR-amplified using high-fidelity DNA polymerase and purified with the TaKaRa MiniBEST Agarose Gel DNA Extraction Kit (Takara, Cat. No. 9762, Tokyo, Japan). Primer sequences are shown in Supplementary Table S2.

### 2.10. T-A Cloning for Identification of Gene Targeting Efficiency

Prepare PCR-amplified knockout cell genome fragments; ligate them with the 18-T (TAKARA Cat. No. 6011, Tokyo, Japan) vector; transform them, followed by colony selection and Sanger sequencing; and pick colonies for low-throughput Sanger sequencing. The targeting efficiency can be calculated by comparing the ratio of the number of sequences with indels to the total number of sequenced sequences using SanpGene (Version 6.2, GSL Biotech LLC, Chicago, IL, USA).

### 2.11. Data Analysis

Data were normalized to the maximum intensity and analyzed with GraphPad Prism 8 (GraphPad Software, San Diego, CA, USA).

## 3. Results

### 3.1. Establishment of the tRNA–sgRNA Array System for Multiplex Gene Editing Using an S/MAR-Based Episomal Targeting Vector

**Figure 1:**
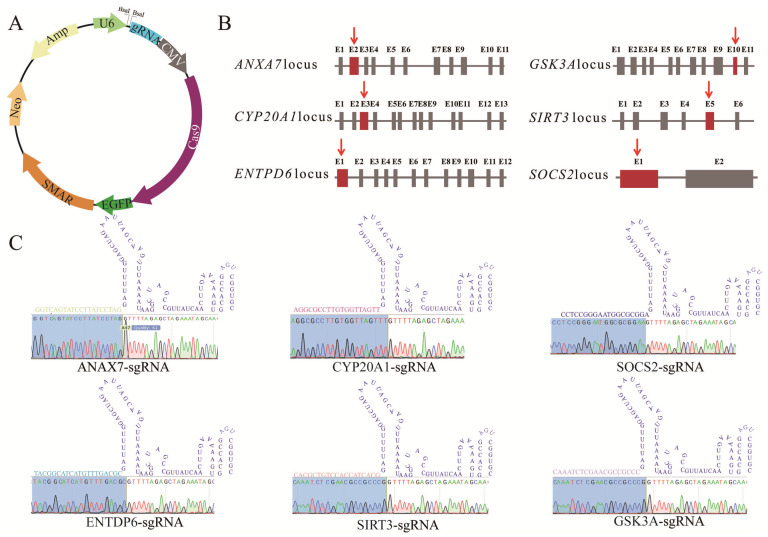
Design of sgRNAs targeting the CDS regions of six porcine genes. (**A**) Schematic diagram of the pCas9GS episomal targeting vector. (**B**) Schematic diagram showing the targeting positions of the sgRNAs designed within the CDS of six selected porcine genes: *SOCS2*, *ENTPD6*, *GSK3A*, *SIRT3*, *ANXA7*, and *CYP20A1*. (**C**) Sequences of the sgRNA corresponding to the red-highlighted region in (**B**), along with predicted secondary structures and representative sequencing results.

The episomal targeting vector constructed in this study comprises four essential functional modules: the sgRNA expression unit, Cas9 coding sequences (CDSs), the S/MAR element, and a G418 resistance cassette. Using PX330 as the backbone, we successfully generated the recombinant vector pCas9GS (PX330-U6-sgRNA-Cas9-GFP-S/MAR-G418) by incorporating elements from the P-EGFP-S/MAR plasmid (providing the S/MAR sequence) and the Cas-G418 plasmid (conferring G418 selection) (Figure 1A). The resulting vector contains the CRISPR targeting module (U6-sgRNA and Cas9 CDS), the S/MAR element, an enhanced green fluorescent protein (EGFP) reporter, and a eukaryotic G418 resistance marker. To validate the efficacy of this vector, we designed sgRNAs targeting six distinct genes. The target loci for each sgRNA are illustrated in Figure 1B, with the protospacer regions highlighted in red. Detailed sequence information for each sgRNA and the corresponding validation data are presented in Figure 1C.

To validate the functional capacity of the episomal targeting vector, we selected six porcine genes (*SOCS2*, *ENTPD6*, *GSK3A*, *SIRT3*, *ANXA7*, and *CYP20A1*) and designed sgRNAs targeting their respective coding sequences (CDSs) (Figure 1C; Supplementary Table S1). For vector construction, each sgRNA sequence was synthesized, subjected to slow annealing to form 20 bp double-stranded DNA fragments with sticky ends, and subsequently ligated into the pCas9GS vector backbone. This yielded six individual knockout constructs: pCas9GS-SOCS2, pCas9GS-ENTPD6, pCas9GS-GSK3A, pCas9GS-SIRT3, pCas9GS-ANXA7, and pCas9GS-CYP20A1.

**Figure 2:**
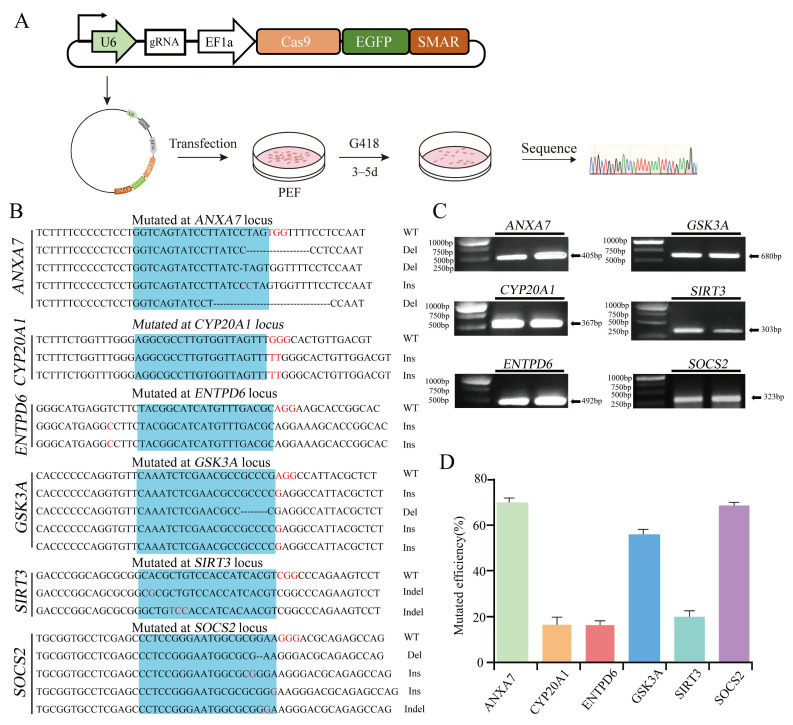
Efficiency of pCas9GS in single-gene targeting of six genes. (**A**) Schematic diagram of the process for verifying targeting efficiency via pCas9GS vector transfection in PEFs. (**B**) Representative Sanger sequencing results showing editing outcomes at each target locus. The protospacer adjacent motif (PAM) is indicated in red; the sgRNA-binding region is shaded in blue. Mutation types are denoted as wild-type (WT), deletion (Del), insertion (Ins), or insertion-deletion (Indel). (**C**) Gel electrophoresis of PCR amplicons for the target gene in the knockout efficiency analysis. Fragment sizes: *GSK3A*, *SIRT3*, *SOCS2*, *ENTPD6*, *ANXA7*, and *CYP20A1*. (**D**) Quantitation of single-gene editing efficiencies determined by Sanger sequencing. Data are presented as the mean ± s.e.m. from three independent experiments.

To evaluate editing efficiency, each targeting vector was transfected into PFFs, and genomic mutations were analyzed by TA cloning, followed by Sanger sequencing (Figure 2A). The PCR amplicon sizes for each target locus were as follows: *GSK3A*, 680 bp; *SIRT3*, 303 bp; *SOCS2*, 323 bp; *ENTPD6*, 492 bp; *ANXA7*, 405 bp; and *CYP20A1*, 367 bp (Figure 2C). Specific primer sequences are listed in Supplementary Table S2. Sequencing results revealed single-gene editing efficiencies of 71.4% for ANXA7-sgRNA1, 16.7% for CYP20A1-sgRNA1, 16.6% for ENTPD6-sgRNA1, 57% for GSK3A-sgRNA1, 20% for SIRT3-sgRNA1, and 70% for SOCS2-sgRNA1 (Figure 2B,D). The frameshift mutation efficiencies were determined as follows: 63.9% for *ANXA7*, 14.08% for *CYP20A1*, 14.72% for *ENTPD6*, 42.75% for *GSK3A*, 17.8% for *SIRT3*, and 56.7% for *SOCS2* (Supplementary Figure S2).

To evaluate editing efficiency, each targeting vector was transfected into PFFs, and genomic mutations were analyzed by TA cloning, followed by Sanger sequencing (Figure 2A). The PCR amplicon sizes for each target locus were as follows: *GSK3A*, 680 bp; *SIRT3*, 303 bp; *SOCS2*, 323 bp; *ENTPD6*, 492 bp; *ANXA7*, 405 bp; and *CYP20A1*, 367 bp (Figure 2C). Specific primer sequences are listed in Supplementary Table S2. Sequencing results revealed single-gene editing efficiencies of 71.4% for ANXA7-sgRNA1, 16.7% for CYP20A1-sgRNA1, 16.6% for ENTPD6-sgRNA1, 57% for GSK3A-sgRNA1, 20% for SIRT3-sgRNA1, and 70% for SOCS2-sgRNA1 (Figure 2B,D). The frameshift mutation efficiencies were determined as follows: 63.9% for *ANXA7*, 14.08% for *CYP20A1*, 14.72% for *ENTPD6*, 42.75% for *GSK3A*, 17.8% for *SIRT3*, and 56.7% for *SOCS2* (Supplementary Figure S2).

### 3.2. Episomal Persistence of S/MAR-Containing Nonviral Vectors in Porcine Fibroblasts

**Figure 3:**
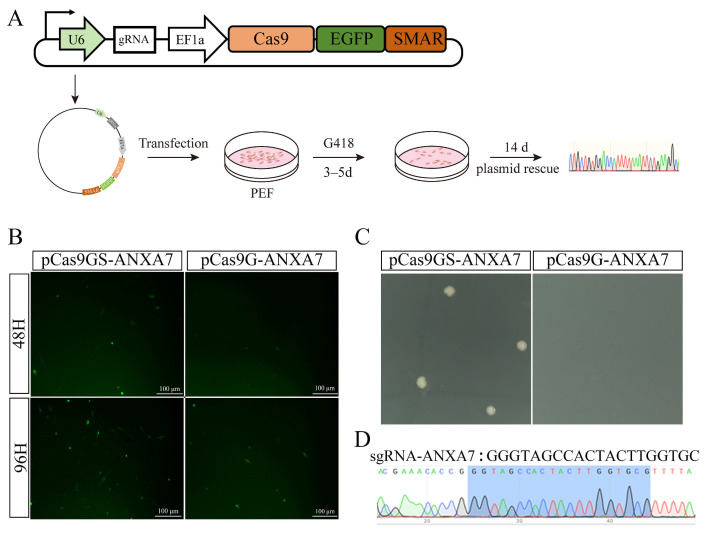
S/MAR element enhances the persistence of green fluorescent plasmids in transfected cells. (**A**) Schematic diagram of the episomal function validation of the pCas9GS plasmid. The episomal maintenance function of the S/MAR element is verified by plasmid rescue experiments after long-term transfection. (**B**) Comparison of fluorescent cell counts at 48 h and 96 h post-transfection. The S/MAR-containing group exhibited significantly higher number of fluorescent cells compared with the S/MAR-deficient group at both time points. (**C**) Plasmid rescue assay results. After 14 days of culture, the target plasmid was successfully recovered from cells transfected with the S/MAR-containing plasmid, whereas no target plasmid was retrieved from the S/MAR-deficient group. (**D**) Sequencing of individual colonies derived from (**C**) verified the structural integrity of the rescued plasmid.

To detect the duration of episomal maintenance and expression, we monitored green fluorescence protein (GFP) expression in cells transfected with vectors either containing or lacking the S/MAR element. PEFs were electroporated with pCas9GS; at 48 h and 96 h post-electroporation, we compared the duration of green fluorescence in cells transfected with green fluorescent plasmids either containing or lacking S/MAR elements (Figure 3A). Notably, the number of fluorescent cells was significantly higher in the S/MAR-containing group compared with the S/MAR-deficient group at the 48 h and 96 h time points (Figure 3B, Supplementary Figure S1), suggesting prolonged episomal retention and sustained expression.

To determine whether the vector exists in a free state within the cells, we performed a plasmid rescue experiment. PEFs were electroporated with either pCas9GS-ANXA7 (S/MAR-containing) or pCas9G-ANXA7 (S/MAR-deficient) and cultured under selection for 14 days. The target plasmid was successfully recovered from cells in the S/MAR-containing group (Figure 3C), indicating that the plasmid pCas9GS-ANXA7 could exist in a free state in the cells for 14 days.

### 3.3. Multiplex Gene Knockout in PFFs Using a tRNA–sgRNA Array Combined with the Episomal CRISPR/Cas9 System

**Figure 4:**
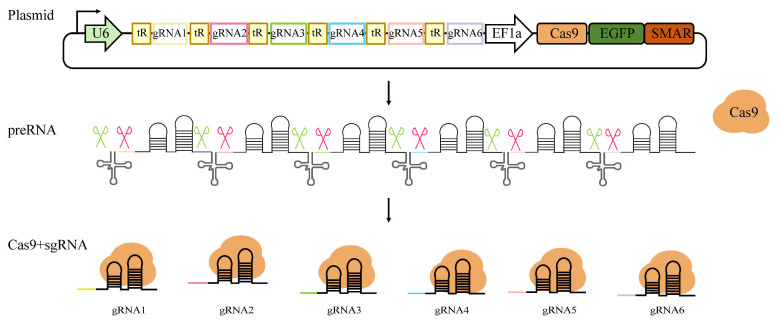
Schematic of multiple sgRNAs’ expression and multi-gene knockout mediated by tRNA self-cleavage. A tRNA–sgRNA array was constructed by tandem arrangement of tRNAs and sgRNAs. Following transcription, tRNA structures within the precursor transcript are recognized and cleaved by endogenous ribozymes, resulting in the release of functional sgRNAs. After binding to these sgRNAs, Cas9 protein can specifically target different genomic loci, enabling synchronous multi-site gene knockout.

To achieve simultaneous expression of multiple sgRNAs, we tandemly arranged sgRNAs with tRNAs to construct a tRNA–sgRNA array. Following transcription, the tRNA moieties within the polycistronic precursor are recognized and cleaved by endogenous RNase P (*Ribonuclease* P) and RNase Z (*Ribonuclease* Z), resulting in the release of individual functional sgRNAs. The Cas9 protein binds to these sgRNAs targeting distinct genomic loci, facilitating synchronous multi-site targeting (Figure 4).

**Figure 5:**
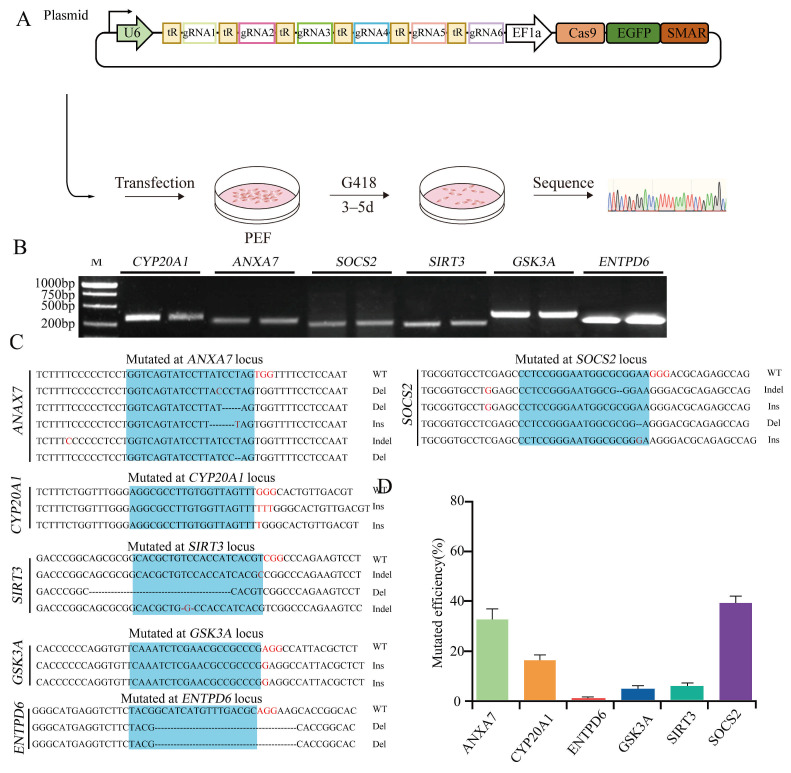
Multi-gene knockout in porcine primary fibroblasts (PEF) using the episomal vector pCas9GS-6sg. (**A**) Flowchart of functional detection of the episomal multi-gene targeting vector. (**B**) Gel electrophoresis results of the amplified fragments of related target genes after successful transfection of the episomal pCas9GS-6sg vector into porcine PEFs. (**C**) Sanger gene sequencing results of the episomal multi-gene co-targeting vector pCas9GS-6sg in porcine PEFs. The PAM is highlighted in red; the sgRNA-binding region is shaded in blue. Mutation types are denoted as wild-type (WT), deletion (Del), insertion (Ins), or insertion–deletion (Indel). (**D**) Quantitative analysis of the simultaneous knockout efficiencies of the six genes (*SOCS2* (40%), *SIRT3* (6%), *GSK3A* (5%), *CYP20A1* (16.6%), *ANXA7* (33.3%), and *ENTPD6* (1.06%)), confirming that this vector can mediate synchronous multi-gene knockout in porcine PEFs. Values represent the mean ± s.e.m. from three independent experiments.

Using the tRNA–sgRNA array technology, we assembled an array encoding six sgRNAs targeting *SOCS2*, *ENTPD6*, *GSK3A*, *SIRT3*, *ANXA7*, and *CYP20A1* and cloned the fragment into the pCas9GS episomal backbone, successfully constructing the episomal multi-gene targeting vector pCas9GS-6sg. A schematic of the vector construction workflow and the results of PCR-amplified fragments are shown in Figure 5A. Sanger sequencing was used to verify the integrity of the sgRNA cassettes. Representative genotyping results are shown in Figure 5B. The observed editing efficiencies for the six genes were as follows: *SOCS2* (40%), *SIRT3* (6%), *GSK3A* (5%), *CYP20A1* (16.6%), *ANXA7* (33.3%), and *ENTPD6* (1.06%) (Figure 5C,D). These results demonstrate that the tRNA-linked multi-gene vector developed in this study can efficiently mediate the knockout of six genes in PFFs.

### 3.4. Simultaneous Knockout of Six Genes in Single-Cell Clones Using an Episomal CRISPR/Cas9 System

**Figure 6:**
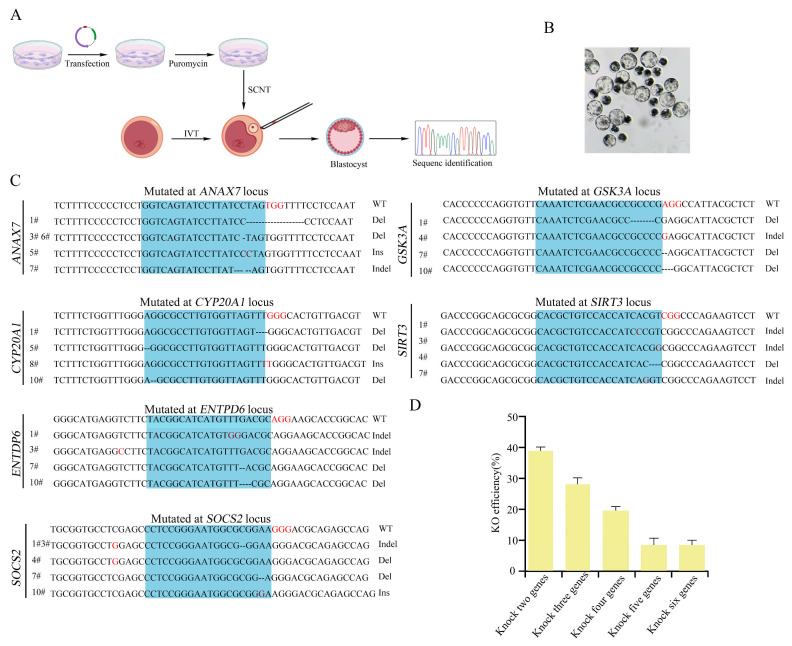
Targeted simultaneous knockout of six genes in a single blastocyst clone. (**A**) Experimental flowchart for verifying the efficiency of multi-gene editing targeting single-cell clones. PFFs were transfected with the pCas9GS-6sg plasmid encoding Cas9 and six tandemly arrayed sgRNAs. Edited cells were used as donor cells for SCNT, and the resulting blastocysts were genotyped by PCR and Sanger sequencing. (**B**) Bright-field images of tRNA-Cas-edited nuclear transfer embryos, showing normal embryonic development. (**C**) Representative Sanger sequencing results from single-blastocyst PCR amplification of the six target gene regions. The PAM site is indicated in red; the sgRNA-binding region is shaded in blue. Mutation types are denoted as wild-type (WT), deletion (Del), insertion (Ins), or insertion-deletion (Indel). (**D**) Quantification of multiplex editing efficiencies in cloned blastocysts. The frequencies of positive clones with concurrent targeting of 2, 3, 4, 5, and 6 genes were 39.09%, 28.20%, 19.09%, 9.09%, and 9.09%, respectively.

To verify the efficacy of the tRNA–sgRNA array strategy in achieving multiplex gene editing within a single cell clone, we transfected PFFs with the pCas9GS-6sg plasmids encoding Cas9 and six tandemly arrayed sgRNAs. Subsequently, the transfected cells were used as donor cells for somatic cell nuclear transfer (SCNT). We assessed embryonic development by quantifying cleavage and blastocyst formation rates (Table 1) and performed genotypic analysis on individual blastocysts (Figure 6A). Notably, the blastocyst development rate of tRNA-Cas-edited cloned embryos showed no significant difference compared with the control group (Figure 6B). DNA fragments of the six target regions were amplified by single blastocyst PCR and subjected to Sanger sequencing (Figure 6C). Since each blastocyst develops from a single donor cell, this experiment allowed the evaluation of the simultaneous knockout efficiency of six genes in individual cells. Sequence analysis revealed that among blastocysts derived from edited donor cells, 39.09% carried edits in two genes, 28.2% in three genes, 19.09% in four genes, 9.09% in five genes, and 9.09% in all six target genes (Figure 6D). These findings demonstrate that the tRNA-Cas strategy enables the generation of cells and embryos carrying multiple gene modifications via a single round of CRISPR-mediated gene editing.

## 4. Discussion

Pigs are a species of considerable importance both in commercial agriculture and as a translational model in biomedical research. Owing to their physiological and genetic similarities to humans, pigs have emerged as an ideal large-animal model for studying human diseases and evaluating therapeutic interventions [1,4]. Genetic modification of pigs through genetic engineering technology may produce humanized pig models for biomedical applications and improve meat production efficiency. For instance, in disease-resistant gene-edited pigs, researchers simultaneously knock out multiple functional genes such as *CD163*, *APN*, and *MSTN* to produce pigs that are both disease-resistant and have improved production performance [2,25]. In the field of xenotransplantation, donor pigs may require the simultaneous modification of up to 10 genes to achieve immunological compatibility [8]. Therefore, the development of efficient multi-gene editing technology in pigs plays a significant role in advancing their use in agriculture and as biomedical models.

Simultaneous editing of multiple genes in PFFs, the donor cells for somatic cell nuclear transfer (SCNT), can substantially reduce the time and effort required to obtain pigs with multiple gene edits. In this study, we proposed a multiplex CRISPR/Cas9 editing system for PFFs based on S/MAR-containing episomal vectors combined with tRNA-gRNA arrays. Using this platform, we attempted the simultaneous knockout of six genes (*ANXA7*, *GSK3A*, *ENTPD6*, *SIRT3*, *CYP20A1*, and *SOCS2*) to generate modified donor cells for SCNT and subsequent production of gene-edited pigs. The selection of these six genes was guided by their reported roles in porcine growth, muscle development, and metabolic regulation. In this study, the editing efficiency presented is highly variable, where some targets show very low efficiency (such as *ENTPD6*, close to ~1%). In this study, all sgRNAs were designed following standardized criteria to ensure high on-target activity and minimize predicted off-target effects, thereby reducing variability attributable to sgRNA design. The remaining differences in editing efficiency are influenced by locus-specific factors, including chromatin accessibility and the local genomic context, which can affect Cas9 access and cleavage efficiency [26]. *ANXA7* is highly expressed in porcine skeletal muscle and modulates the calcium signaling pathway in muscle cells, potentially affecting muscle fiber type and growth rate [27,28]. Polymorphisms in *GSK3A* are significantly associated with lean meat percentage, backfat thickness, and loin muscle area; systemic knockout of Gsk3a in mice leads to increased body weight, lean mass, reduced fat [29], improved insulin sensitivity, and glucose tolerance [30]. *ENTPD6* is expressed in porcine skeletal muscle and adipose tissue, affecting muscle fiber type, intramuscular fat, and growth rate through glycosylation and energy metabolism [24,27]. *SIRT3* regulates muscle cell differentiation, muscle fiber type, and energy efficiency, influencing growth rate, lean meat percentage, and eye muscle area [31,32]. *CYP20A1* is expressed in muscle and fat, and is thought to influence growth hormone secretion, muscle fiber development, and fat deposition through the neuro-endocrine axis; it may affect growth rate, lean meat percentage, and intramuscular fat [33]. Additionally, it regulates the stress axis (HPA), oxidative stress, and neurobehavior and is associated with stress resistance, social behavior, and breeding stability [33]. Finally, *SOCS2* is located in the feed conversion rate quantitative trait locus (QTL) interval on chromosome 5, and its SNPs are significantly associated with daily gain, feed-to-gain ratio, and eye muscle area [34,35]. Low expression or functional loss of this gene results in faster growth, lower feed-to-gain ratio, and higher lean meat percentage [34,35]. We hypothesized that simultaneous disruption of these six genes in pigs could yield synergistic phenotypes, including enhanced muscle growth and accelerated developmental rates. The work presented here provides a foundation for generating multi-gene-edited pigs and demonstrates the utility of multiplex genome editing for both agricultural improvement and the development of advanced porcine models.

Multiplex genome editing is an intriguing advantage of CRISPR/Cas9 technology, which can be achieved by simultaneously expressing multiple gRNAs. The gRNA–tRNA array (GTR) system enables the expression of up to 31 sgRNAs from a single transcript, and by facilitating multi-locus targeting using a single vector, this approach holds significant promise for advancing high-throughput CRISPR/Cas9-based genomic research [36].The tRNA-based multiplex sgRNA systems enable efficient multi-gene knockout, highlighting the broad applicability of CRISPR-based multiplex editing across species, including human, mouse, zebrafish, and crops [12,13]. Notably, Pol II promoters can express the GTR system and generate multiple gRNAs from a single transcript [11]. Inducible and tissue-specific promoters can be used to explore complex interactions among diverse regulatory signals [11].

Episomal vectors offer multiple advantages over integrative systems. They can stably express CRISPR components without integrating exogenous genes into the host genome, thereby reducing the risk of insertional mutations and disruption of essential genes. S/MAR mediates binding to the nuclear matrix, which not only extends the intracellular survival time of the vector but also promotes episomal replication of the plasmid [19]. Importantly, S/MAR sequences can drive the retention of episomal plasmids without expressing any proteins. Previous studies have demonstrated the utility of S/MAR-based vectors for nonviral gene delivery, including stable labeling of cells for in vivo tracking and sustained expression of therapeutic transgenes [19]. For example, S/MAR vectors have been used to efficiently transfect and modify primary human T cells carrying chimeric antigen receptor (CAR) constructs [37], and have supported transgene expression in induced pluripotent stem cells (iPSCs) and mouse embryonic stem cells for up to 170 days [38]. Transcriptomic analyses have further shown that S/MAR vector delivery exerts minimal impact on the host cell’s transcriptome [39]. For CRISPR/Cas9 gene editing, the episomal vector systems enable efficient genome editing with sustained long-term expression, while maintaining low off-target effects even during extended application, thereby ensuring their safety and suitability for research purposes [40]. Distinct from these prior applications, the present study represents a novel application of S/MAR vectors as a tool to facilitate gene editing, rather than for delivering therapeutic transgenes. This new direction introduces a distinct set of challenges and objectives, expanding the functional repertoire of episomal vector technology in genome engineering.

Due to the difficulty in identifying the efficiency of multiple gene knockouts in a single cell, we determined the simultaneous knockout of six genes in a single cell clone by identifying the genotype of the blastocyst. The blastocyst was generated using somatic cell nuclear transfer (SCNT) technology, with gene-edited PEFs as the donor cells. Our results indicate that the GTR-CRISPR system enabled the concurrent induction of mutations within the coding regions of *ANXA7*, *GSK3A*, *ENTPD6*, *SIRT3*, *CYP20A1*, and *SOCS2* in a single blastocyst, confirming that this multiplex gene editing strategy supports simultaneous modification of six genes in a single cell. Notably, a similar approach has been reported to achieve successful targeting of up to eight genes in Saccharomyces cerevisiae, suggesting that designing GTRs can also target more genes in pigs [36]. In the present study, our primary objective was to evaluate whether a single vector enables the simultaneous editing of multiple target genes. Accordingly, off-target effects were not systematically analyzed in this work. A comprehensive assessment of off-target efficiency will be conducted in future investigations.

## 5. Conclusions

In summary, the episomal multi-gene CRISPR editing vector described herein enables the simultaneous expression of six sgRNAs and induces targeted disruptions within the coding sequences of *ANXA7*, *GSK3A*, *ENTPD6*, *SIRT3*, *CYP20A1*, and *SOCS2* in PFFs. The method based on the episomal vector multi-gene editing system achieves multi-gene editing by simply transfecting one vector, which simplifies the process and enhances the efficiency of CRISPR/Cas9 multi-gene editing in pigs. Looking forward, this strategy based on the combination of episomal vectors and tRNA–sgRNA systems holds promise for widespread adoption in multiplex genome engineering in porcine models and beyond.

## Figures and Tables

**Table 1:** The development of GTR-cloned embryos in vitro.

Groups	Embryos	No. (%) of Cleavages	No. (%) of Blastocysts
GTR	152	123 (81 ± 1.9)	20 (13.1 ± 2.1)
control	149	125 (83.8 ± 2.1)	20 (13.4 ± 2.0)

## Data Availability

The datasets used and/or analyzed during the current study are available from the corresponding author on reasonable request.

## Supplementary Materials

The following supporting information can be downloaded at: https://www.mdpi.com/article/10.3390/biology15100742/s1, Supplementary Figure S1. Comparison of the number of fluorescent foci between plasmids with and without S/MAR elements. Supplementary Figure S2. Quantitative analysis of frameshift mutation efficiencies for the six target genes. Supplementary Table S1: Information of the sgRNA. Supplementary Table S2: Primers for target gene fragment amplification.

## References

[1] Ju W.S., Kim S., Lee J.Y., Lee H., No J., Lee S., Oh K. (2025). Gene Editing for Enhanced Swine Production: Current Advances and Prospects. Animals.

[2] Xu K., Zhou Y., Mu Y., Liu Z., Hou S., Xiong Y., Fang L., Ge C., Wei Y., Zhang X. (2020). CD163 and pAPN double-knockout pigs are resistant to PRRSV and TGEV and exhibit decreased susceptibility to PDCoV while maintaining normal production performance. eLife.

[3] Khan N., Li Z., Ali A., Quan B., Kang J., Ullah M., Yin X.J., Shafiq M. (2025). Comprehensive transcriptomic analysis of myostatin-knockout pigs: Insights into muscle growth and lipid metabolism. Transgenic Res..

[4] Lunney J.K., Van Goor A., Walker K.E., Hailstock T., Franklin J., Dai C. (2021). Importance of the pig as a human biomedical model. Sci. Transl. Med..

[5] Wang J.Y., Doudna J.A. (2023). CRISPR technology: A decade of genome editing is only the beginning. Science.

[6] Ran F.A., Hsu P.D., Wright J., Agarwala V., Scott D.A., Zhang F. (2013). Genome engineering using the CRISPR-Cas9 system. Nat. Protoc..

[7] Cao J., Wu L., Zhang S.M., Lu M., Cheung W.K., Cai W., Gale M., Xu Q., Yan Q. (2016). An easy and efficient inducible CRISPR/Cas9 platform with improved specificity for multiple gene targeting. Nucleic Acids Res..

[8] Duan X., Chen C., Du C., Guo L., Liu J., Hou N., Li P., Qi X., Gao F., Du X. (2025). Homozygous editing of multiple genes for accelerated generation of xenotransplantation pigs. Genome Res..

[9] Hong L., Zhang C., Jiang Y., Liu H., Huang H., Guo D. (2020). Therapeutic status and the prospect of CRISPR/Cas9 gene editing in multiple myeloma. Future Oncol..

[10] Hung S.W., Chuang C.K., Wong C.H., Yen C.H., Peng S.H., Yang C., Chen M.C., Yang T.S., Tu C.F. (2023). Activated macrophages of CD 163 gene edited pigs generated by direct cytoplasmic microinjection with CRISPR gRNA/Cas9 mRNA are resistant to PRRS virus assault. Anim. Biotechnol..

[11] McCarty N.S., Graham A.E., Studena L., Ledesma-Amaro R. (2020). Multiplexed CRISPR technologies for gene editing and transcriptional regulation. Nat. Commun..

[12] Shiraki T., Kawakami K. (2018). A tRNA-based multiplex sgRNA expression system in zebrafish and its application to generation of transgenic albino fish. Sci. Rep..

[13] Abdelrahman M., Wei Z., Rohila J.S., Zhao K. (2021). Multiplex Genome-Editing Technologies for Revolutionizing Plant Biology and Crop Improvement. Front. Plant Sci..

[14] Campa C.C., Weisbach N.R., Santinha A.J., Incarnato D., Platt R.J. (2019). Multiplexed genome engineering by Cas12a and CRISPR arrays encoded on single transcripts. Nat. Methods.

[15] Cheng H., Zhang F., Ding Y. (2021). CRISPR/Cas9 Delivery System Engineering for Genome Editing in Therapeutic Applications. Pharmaceutics.

[16] Du Y., Liu Y., Hu J., Peng X., Liu Z. (2023). CRISPR/Cas9 systems: Delivery technologies and biomedical applications. Asian J. Pharm. Sci..

[17] Argyros O., Wong S.P., Harbottle R.P. (2011). Non-viral episomal modification of cells using S/MAR elements. Expert Opin. Biol. Ther..

[18] Wasag P., Lenartowski R. (2016). Nuclear matrix—Structure, function and pathogenesis. Postep. Hig. Med. Dosw. (Online).

[19] Verghese S.C., Goloviznina N.A., Skinner A.M., Lipps H.J., Kurre P. (2014). S/MAR sequence confers long-term mitotic stability on non-integrating lentiviral vector episomes without selection. Nucleic Acids Res..

[20] Athanassiadou A., Sgourou A., Verras M. (2025). The beta-Hemoglobinopathies as a Model for the Development of Nonviral, Episomal Vectors for Gene Therapy. Hum. Gene Ther..

[21] Papapetrou E.P., Ziros P.G., Micheva I.D., Zoumbos N.C., Athanassiadou A. (2006). Gene transfer into human hematopoietic progenitor cells with an episomal vector carrying an S/MAR element. Gene Ther..

[22] Lazaris V.M., Simantirakis E., Stavrou E.F., Verras M., Sgourou A., Keramida M.K., Vassilopoulos G., Athanassiadou A. (2023). Non-Viral Episomal Vector Mediates Efficient Gene Transfer of the beta-Globin Gene into K562 and Human Haematopoietic Progenitor Cells. Genes.

[23] Toualbi L., Toms M., Almeida P.V., Harbottle R., Moosajee M. (2023). Gene Augmentation of CHM Using Non-Viral Episomal Vectors in Models of Choroideremia. Int. J. Mol. Sci..

[24] Turcot V., Lu Y., Highland H.M., Schurmann C., Justice A.E., Fine R.S., Bradfield J.P., Esko T., Giri A., Graff M. (2018). Protein-altering variants associated with body mass index implicate pathways that control energy intake and expenditure in obesity. Nat. Genet..

[25] Wang Y., Bi D., Qin G., Song R., Yao J., Cao C., Zheng Q., Hou N., Wang Y., Zhao J. (2020). Cytosine Base Editor (hA3A-BE3-NG)-Mediated Multiple Gene Editing for Pyramid Breeding in Pigs. Front. Genet..

[26] Shalem O., Sanjana N.E., Hartenian E., Shi X., Scott D.A., Mikkelson T., Heckl D., Ebert B.L., Root D.E., Doench J.G. (2014). Genome-scale CRISPR-Cas9 knockout screening in human cells. Science.

[27] Wu Z., Wang Z., Wang P., Cheng L., Li J., Luo Y., Yang L., Li L., Zeng J., Hu B. (2024). Integrative analysis of proteomics and lipidomic profiles reveal the fat deposition and meat quality in Duroc x Guangdong small spotted pig. Front. Vet. Sci..

[28] Chen L., Liu H., Jiang L., Wang Z., Chang Y., Li N., Feng S. (2025). Lipid Droplets Metabolism Mediated by ANXA7-PPARgamma Signaling Axis Regulates Spinal Cord Injury Repair in Mice. Adv. Sci..

[29] Fu Y., Li C., Tang Q., Tian S., Jin L., Chen J., Li M., Li C. (2016). Genomic analysis reveals selection in Chinese native black pig. Sci. Rep..

[30] Bali S.K., Bryce D., Prein C., Woodgett J.R., Beier F. (2021). Glycogen synthase kinase 3 alpha/beta deletion induces precocious growth plate remodeling in mice. J. Mol. Med..

[31] Coelho D., Ribeiro D., Osorio H., de Almeida A.M., Prates J.A.M. (2022). Integrated Omics analysis of pig muscle metabolism under the effects of dietary Chlorella vulgaris and exogenous enzymes. Sci. Rep..

[32] Song H., Thompson L.P. (2023). Effects of Gestational Hypoxia on PGC1alpha and Mitochondrial Acetylation in Fetal Guinea Pig Hearts. Reprod. Sci..

[33] Chen L., Chen L., Li X., Qin L., Zhu Y., Zhang Q., Tan D., He Y., Wang Y.H. (2022). Transcriptomic profiling of hepatic tissues for drug metabolism genes in nonalcoholic fatty liver disease: A study of human and animals. Front. Endocrinol..

[34] Chen Y., Piper E., Zhang Y., Tier B., Graser H.U., Luxford B.G., Moran C. (2011). A single nucleotide polymorphism in suppressor of cytokine signalling-2 is associated with growth and feed conversion efficiency in pigs. Anim. Genet..

[35] Yang H.L., Sun C., Sun C., Qi R.L. (2012). Effect of suppressor of cytokine signaling 2 (SOCS2) on fat metabolism induced by growth hormone (GH) in porcine primary adipocyte. Mol. Biol. Rep..

[36] Zhang Y., Wang J., Wang Z., Zhang Y., Shi S., Nielsen J., Liu Z. (2019). A gRNA-tRNA array for CRISPR-Cas9 based rapid multiplexed genome editing in Saccharomyces cerevisiae. Nat. Commun..

[37] Llanos-Ardaiz A., Lantero A., Neri L., Mauleón I., Ruiz de Galarreta M., Trigueros-Motos L., Weber N.D., Ferrer V., Aldabe R., Gonzalez-Aseguinolaza G. (2024). In Vivo Selection of S/MAR Sequences to Favour AAV Episomal Maintenance in Dividing Cells. Int. J. Mol. Sci..

[38] O’Doherty R., Greiser U., Wang W. (2013). Nonviral Methods for Inducing Pluripotency to Cells. BioMed Res. Int..

[39] Hartley A., Burger L., Wincek C.L., Dons L., Li T., Grewenig A., Taşgın T., Urban M., Roig-Merino A., Ghazvini M. (2024). A Simple Nonviral Method to Generate Human Induced Pluripotent Stem Cells Using SMAR DNA Vectors. Genes.

[40] Duan N., Tang S., Zeng B., Hu Z., Hu Q., Wu L., Zhou M., Liang D. (2021). An Episomal CRISPR/Cas12a System for Mediating Efficient Gene Editing. Life.

