# Supplementary material for: An Episomal Clustered Regularly Interspaced Short Palindromic Repeats/Cas9 System for Transgene-Free Multiplex Gene Editing in Pig Cells
Source: Biology (Basel). 2026 May 8;15(10):742. doi: 10.3390/biology15100742 (PMC13203174; doi:10.3390/biology15100742)
Supplement: Supplementary file 1 [file biology-15-00742-s001.zip › biology-4248658-supplementary.pdf]

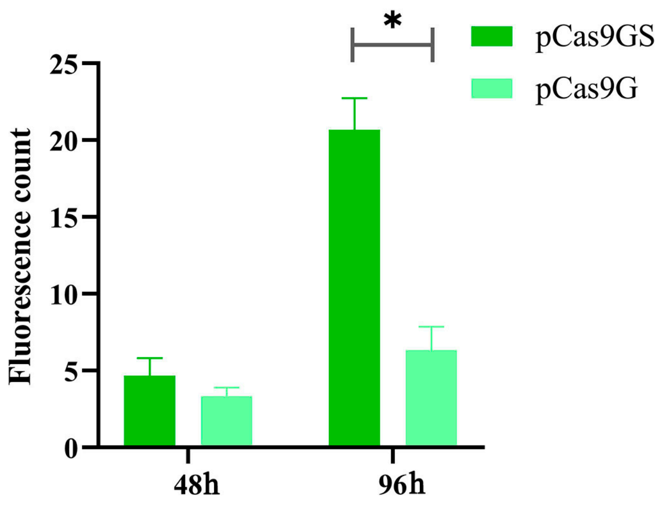

Supplementary Figure S1. Comparison of the number of fluorescent foci between plasmid with and without S/MAR elements.

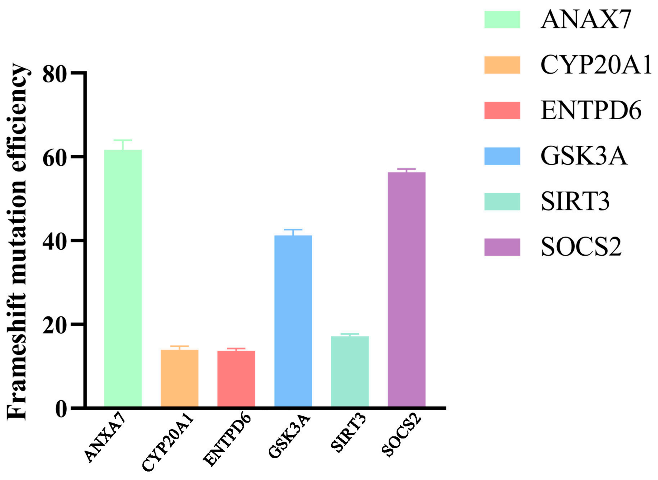

Supplementary Figure S2. Quantitative analysis of frameshift mutation efficiencies for six target genes.

Supplementary Table S1 Information of the sgRNA

Gene	sgRNA sequence (5’-3’)	PAM	GC%
SIRT3	CACGCTGTCCACCATCACGT	CGG	60
ENTPD6	TACGGCATCATGTTTGACGC	AGG	50
SOCS2	CCTCCGGGAATGGCGCGGAA	GGG	70
CYP20A1	AGGCGCCTTGTGGTTAGTTT	GGG	50
ANXA7	GGTCAGTATCCTTATCCTAG	TGG	45
GSK3A	CAAATCTCGAACGCCGCCCG	AGG	50

Supplementary Table S2 Primers for target gene fragment amplification

Gene	Primer sequence (5’-3’)
SOCS2-S	GGATTCGTTTTGGGGTTCT
SOCS2-A	CAGCCTCGCCAGCACAG
ANXA7-S	AAAAGCTAGACCTTAGCAGTTATCTCTGTTTGTTTCCTT TTTTAGAAGGATTATGTTTC
ANXA7-A	GAAGTGGAAAGTAGCCTAAGTGCAACTCTGACCTCAT ACAAAACAAGAAGAGGATTAAA
CYP20A1-S	TCTCATCTGTTGATACAGCTAGTTCAGAATCAGCTAAT ATTTTTTTTCTGACTCTCCCA
CYP20A1-A	AATGTTAGCCATTGCTATAAGAACAATGTATGGTCTTC CTTTTTGGATATTTATTCTTA
GSK3A-S	GAGCCAGACAGAAACTCCCAAGA
GSK3A-A	AGAGCCTAGCACAAGACATCACAGA
GSK3A-S-I	CTCACCTGATACAAACTGACCTCCTC
GSK3A-A-I	TTGCCTCGTAGCCCACCCTC
SIRT3-S	GAGTTTTCCCTTTTCCCCTAA
SIRT3-A	AGCCCCGCCTGCTTT
ENTDP6-S	GATTTCTGACTCGCCCTGGAACCTCGCAGCCTAACGGG TTTTTCCTGCCTCTCCTCCCT
ENTDP6-A	CTCTGACAGGCTCCCAGGTCCAAGCCAGCGCAAGGCA GGGGGTCCAACGCACAGCCCCA
ENTDP6-S-I	GGGCTGTTGGTGTGCCT
ENTDP6-A-I	CAGACAACAGTGAGAAGCGT
